# Preoperative hemoglobin and perioperative blood transfusion in major head and neck surgery: a systematic review and meta-analysis

**DOI:** 10.1186/s40463-022-00588-4

**Published:** 2023-01-24

**Authors:** Munib Ali, Joseph C. Dort, Khara M. Sauro

**Affiliations:** 1 Cumming School of Medicine, University of Calgary, 3280 Hospital Dr. NW, Room 3D41, Calgary, AB T2N 4Z6 Canada; 2 Ohlson Research Initiative, University of Calgary, Calgary, AB Canada; 3 Department of Community Health Sciences, University of Calgary, Calgary, AB Canada; 4 Department of Oncology, University of Calgary, Calgary, AB Canada; 5 Department of Surgery, University of Calgary, Calgary, AB Canada

**Keywords:** Head and neck surgery, Free flap reconstruction, Preoperative anemia, Blood transfusion, Systematic review, Meta-analysis, Otolaryngology, Otorhinolaryngology, ENT, Ears nose throat, Head and neck cancer, Head and neck carcinoma, Head and neck neoplasms, Free tissue transfer

## Abstract

**Background:**

There is a growing concern with inappropriate, excessive perioperative blood transfusions. Understanding the influence of low preoperative hemoglobin (Hgb) on perioperative blood transfusion (PBT) in head and neck cancer (HNC) surgery with free flap reconstruction may help guide clinical practice to reduce inappropriate treatment among these patients. The objective is to synthesize evidence regarding the association between preoperative Hgb and PBT among major HNC free flap surgeries.

**Methods:**

Terms and synonyms for HNC surgical procedures, Hgb and PBT were used to search MEDLINE, Embase, CINAHL, Cochrane Central Register of Controlled Trials and Cochrane Database of Reviews from inception to February 2020. Reference lists of included full texts and studies reporting the preoperative Hgb, anemia or hematocrit (exposure) and the PBT (outcome) in major HNC surgery with free flap reconstruction were eligible. Studies examining esophageal, thyroid and parathyroid neoplasms were excluded; as were case reports, case series (n < 20), editorials, reviews, perspectives, viewpoints and responses. Two independent, blinded reviewers screened titles, abstracts and full texts in duplicate. The Preferred Reporting Items for Systematic Reviews and Meta-Analyses was followed. A random-effects model was used to pool reported data. The primary outcome was the proportion of patients who had a PBT. Subgroup analysis examined sources of heterogeneity for perioperative predictors of PBT (age, sex, flap type, flap site and preoperative Hgb). We also examined mean preoperative Hgb in the PBT and no PBT groups.

**Results:**

Patients with low preoperative Hgb were transfused more than those with normal Hgb (47.62%, 95% CI = 41.19–54.06, I^2^ = 0.00% and 13.92%, 95% CI = 10.19–17.65, I^2^ = 20.69%, respectively). None of the predictor variables explained PBT. The overall pooled mean preoperative Hgb was 12.96 g/dL (95% CI = 11.33–14.59, I^2^ = 0.00%) and was 13.58 g/dL (95% CI = 11.95–15.21, I^2^ = 0.00%) in the no PBT group and 12.05 g/dL (95% CI = 10.01 to 14.09, I^2^ = 0.00%) in the PBT group.

**Conclusions:**

The heterogeneity between studies, especially around the trigger for PBT, highlights the need for additional research to guide clinical practice of preoperative Hgb related to PBT to enhance patient outcomes and improve healthcare stewardship.

**Graphical abstract:**

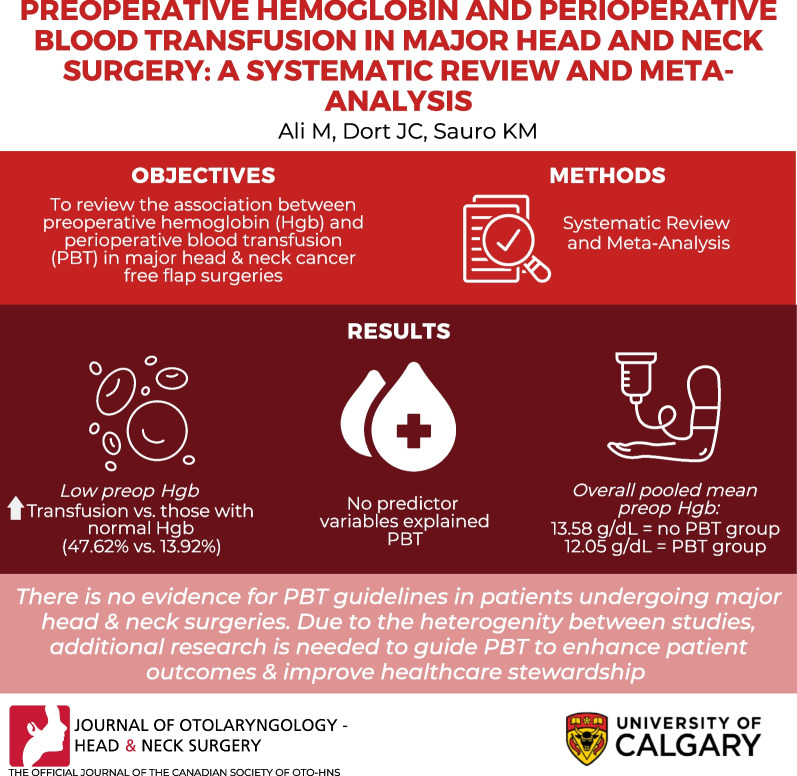

**Supplementary Information:**

The online version contains supplementary material available at 10.1186/s40463-022-00588-4.

## Background

Head and neck cancer (HNC) refers to malignant neoplasms found in the upper aerodigestive tract and is the seventh most common type of cancer worldwide [1]. The 5-year survival for HNC ranges from approximately 33% to 97% [2]. Tobacco, alcohol, human papillomaviruses, and Epstein-Barr virus are related to the pathogenesis of head and neck squamous cell carcinoma (HNSCC)—the most common subtype of HNC [3–5].

Surgical resection is a mainstay treatment for HNSCC and is often lengthy, complex, and frequently removes structures that are important for speaking, swallowing and breathing. Reconstruction of these critical structures is therefore a key component of the surgical plan in most major HNC surgeries. Free flap surgery is the most common approach to restore resected tissue and allow functional recovery after major HNC surgery. The multiple donor operative sites in free flap surgeries increases the duration and complexity of major HNC surgery, which consequently results in frequent complications. Therefore, prior to surgery, identifying modifiable factors that predispose patients to complications is important.

Blood loss is a common complication of HNC free flap surgery which may lead to perioperative blood transfusion (PBT). PBT is a form of tissue transplant that increases the risk of transfusion adverse reactions, infection, transfusion-related acute lung injury, multi-organ dysfunction syndrome, immunomodulation, and recurrent cancer [6–8]. In HNC free flap surgery, ample literature demonstrates the association of PBT with tumor recurrence and lower overall survival [9–18]. In a cohort of 282 HNC free flap procedures, PBT was not only associated with greater length of stay, but also a range of medical complications such as myocardial infarction, congestive heart failure, pneumonia, and respiratory distress [19]. The sequelae of PBT are associated with the volume of blood transfused; the use of 3 units or more is associated with poorer recurrence-free survival and overall survival [11]. Conversely, timing of PBT is not associated with some outcomes, such as flap outcomes [20].

For surgical patients with preoperative anemia, intraoperative blood loss will further lower blood hemoglobin (Hgb) concentrations. PBT is used in these cases to manage severe anemia to improve tissue perfusion and free flap oxygenation. As such, low preoperative hemoglobin Hgb is an important prognostic factor for PBT and predicts increased PBT in a dose-dependent manner [11, 21]. Preoperative anemia is a modifiable risk factor that increases the use of PBT. In HNC patients, anemia may be caused by iron deficiency, chronic disease, prior treatment, or other factors [22–24]. Anemia is defined by the World Health Organization as a Hgb concentration below 13 g/dL in adult males and below 12 g/dL in adult, non-pregnant females [25]. Preoperative anemia is independently related to postoperative complications. In a study of 227,425 subjects investigating the effects of preoperative anemia on postoperative outcomes in noncardiac surgery, it was found that preoperative anemia independently increases the risk of 30-day morbidity and mortality [26]. Anemia independently contributes to an increase in the relative risk of death by 75% in HNC surgery [27].

The costs and potential risks of PBT has led to a reevaluation of blood transfusion practices. Correcting anemia prior to surgery may not only reduce the need for PBT, but also the complications associated with low preoperative Hgb. The purpose of this meta-analysis is to review and summarize the current literature in major HNC free flap surgery on the association between preoperative Hgb and PBT. This information could inform clinicians about appropriate transfusion triggers and help to preoperatively identify patients at high risk of needing PBT. Early identification of high-risk patients could potentially enable preoperative anemia therapy and mitigate the need for PBT.

## Methods

This review was registered with the Prospective Register of Systematic Reviews (PROPSERO, CRD42020165924). This systematic review and meta-analysis was conducted in accordance with the preferred reporting items for systematic reviews and meta-analyses (PRISMA) [28].

### Information sources and search strategy

The search terms used to identify evidence sources contained a comprehensive list of subject headings, keywords, structured language and synonyms related to surgical procedures, neoplasms and anatomy of the head and neck, anemia and blood transfusion (Additional file 1: Appendix A). The terms were reviewed by the research team including a head and neck surgeon (JCD) and a research librarian with expertise in systematic reviews. The search terms were run in MEDLINE, Embase, Cochrane Central Register of Controlled Trials (CENTRAL) and Cochrane Database of Systematic Reviews using Ovid and in CINAHL using EBSCOhost. To minimize publication bias, we searched the grey literature, specifically in clinicaltrials.gov and websites of otolaryngology, surgery, anesthesiology and transfusion medicine related organizations and societies. All databases were searched simultaneously from inception to February 3, 2020. To identify any additional references, manual searching, “cited by” features, PubMed’s “suggested articles” feature and the reference section of identified studies were searched.

### Eligibility criteria

Studies were eligible for inclusion if they studied adults (18 years of age or older) undergoing major HNC free flap surgery. Major HNC surgery was defined as tumor resection (plus or minus neck dissection) with free flap reconstruction. Thyroid, parathyroid and esophageal neoplasms were excluded. Studies examining both the exposure (preoperative Hgb, hematocrit or anemia) and the outcome (PBT) variables were included. Case reports, case series (n < 20), editorials, perspectives, scoping or literature reviews, and responses were excluded. Both journal articles and conference proceedings were included.

### Study selection

The software *Covidence* was used for study selection and data management [29]. The first 20 references were reviewed by all reviewers to ensure there was consistent application of the eligibility criteria. Once the reviewers were satisfied with consistency of the review process, the reviewers independently reviewed of all references in duplicate. All references required at least one of two votes to be considered for full-text review (without resolving conflict). A similar approach was used for full-text review. All reviewers screened the same 10 articles to ensure consistent application of the eligibility criteria and reasons for exclusion. Agreement between the two independent reviewers was required to determine eligibility of the studies and to determine the reason for exclusion. All inconsistencies were discussed and resolved via consensus for the final inclusion. The corresponding authors of studies and abstracts thought to have relevant data outside of what was reported, were contacted.

### Data extraction and quality appraisal

A standardized data extraction tool was developed in *Excel* (Additional file 2: Appendix B)*.* One reviewer abstracted data, which was then reviewed by a second reviewer. Bibliographic information of the included studies and demographic data of the samples were abstracted. The outcome variables of interest were separated into three categories: (1) the **exposure** variable included the preoperative hemoglobin values, hematocrit values or indications of anemia, (2) the **outcome** variable included the occurrence of blood transfusions and the number of units of blood transfused (if indicated), and (3) potential **predictor variables** included: age, sex, ethnicity, comorbidities, American Society of Anesthesiologists Class, tumour site, cancer staging, intraoperative blood loss, history of bleeding disorders, history of smoking, history of alcohol consumption, radiation, chemotherapy, blood transfusion trigger point, body mass index, length of surgery, concurrent procedures and type of free flap used. Any hematocrit values (Hct, ie. transfusion trigger points) were converted to Hgb by dividing the Hct by three. All study details were also recorded. Authors were contacted by email for additional information where necessary.

The Newcastle–Ottawa Quality Assessment for Cohort Studies was used to evaluate the risk of bias within studies [30]. The Newcastle–Ottawa examines the risk of bias related to selection, comparability and outcome measures [30]. A final rating was the proportion of the number of stars (points) given in each domain over the total possible number of points. Two reviewers independently appraised the quality of the included studies.

### Statistical analysis

All statistical analyses were conducted in *Stata 14.0* [31]. If it was appropriate to pool estimates (i.e., comparable data in at least two studies), it was determined a priori that a random effects model for meta-analysis be employed. Sources of heterogeneity between estimates were explored; specifically, we investigated the heterogeneity in the pooled proportion of PBT attributed to preoperative variables (age, sex, flap type, flap site, tumour stage, node involvement, clinical cancer stage, neck dissection, and preoperative Hgb). The I^2^ was used to quantify the heterogeneity between studies and the Cochrane Q statistic was used to determine the statistical significance of I^2^. A 95% confidence interval was used to report the estimates. To evaluate publication bias in this review, a funnel plot was generated and assessed visually and graphically using Egger’s linear regression. An alpha of 0.05 was used as the threshold for statistical significance.

## Results

**Fig. 1:**
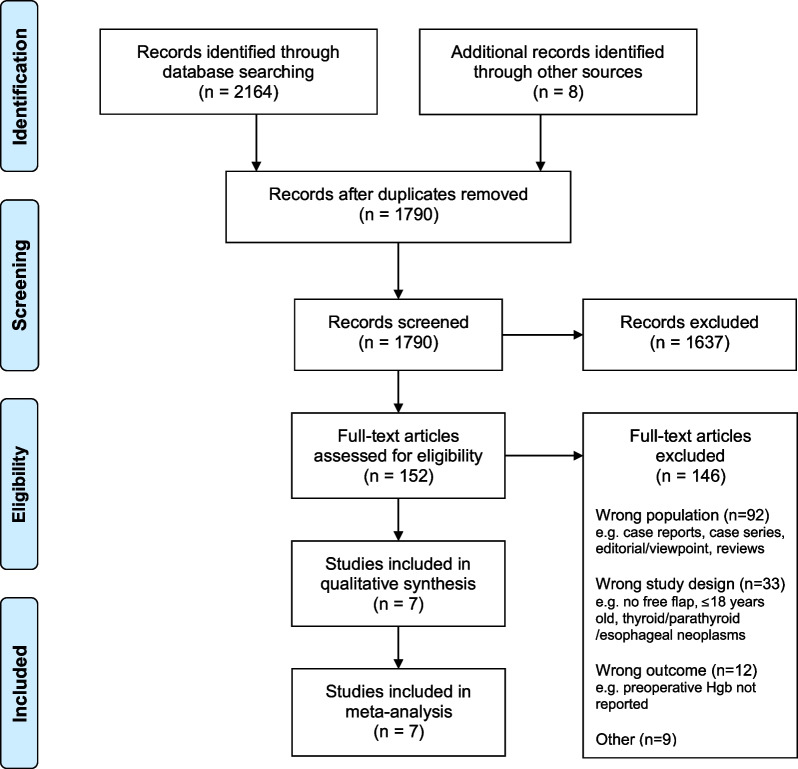
PRISMA flow diagram

The initial search identified 2 164 studies. After the removal of duplicates, 1 782 references were screened at the title and abstract phase and 144 data sources were screened in full text. An additional eight studies were identified from manual searches through reference lists and the web. Six journal articles and one conference abstract met the eligibility criteria for inclusion upon the completion of the full text review stage [11, 21, 32–36]. Three of the seven studies provided additional data upon request [33, 34, 36]. The PRISMA flow diagram shows the flow of studies through the review (Fig. 1).

Table 1 summarizes the overall bibliographic and demographic characteristics of the included studies. Briefly, our study represents data from 1 191 patients from five different countries. All included studies were retrospective cohort studies conducted between 2010 and 2019. The mean number of patients per study was 170 (range = 64–585) and 66.5% of the patients were male. Patients presenting with low Hgb ranged from 19 to 55%. The definition of anemia or low Hgb in the studies varied; one study used a modified classification [32], two studies did not report a classification approach [11, 35] and four studies adhered to WHO classification [21, 33, 34, 36] (below 13 g/dL in males and below 12 g/dL in non-pregnant females) [25]. Transfusion triggers varied between centers; one center performed transfusions at 7 g/dL [33], another center at 8-9 g/dL [34], yet another center used 10 g/dL [11], two centers transfused at the discretion of the surgeon or anesthesiologist [32, 36], and finally, two centers did not report the transfusion trigger [21, 35].

**Table 1:** Summary of the study and demographic variables of the included studies

Author	Year	n	Male n (%)^a^	Age ≥ 60 n (%)^a^	Osseous flap n (%)^a^	Low Hgb n (%)	PBT n (%)	Anemia/low Hgb Males (g/dL)	Anemia/low Hgb Females (g/dL)	PBT trigger (g/L)
Shah	2010	585	309 (67)	308 (53)	105 (18)	160 (27)	144 (25)	≤ 130	≤ 120	
Perisanidis	2013	142	104 (73)	45 (32)	41 (29)	66 (46)	121 (85)	≤ 120	≤ 120	
Abu-Ghanem^b^	2015	65	44 (68)	40 (62)		18 (28)	14 (22)	≤130	≤ 120	
Danan	2015	167	114 (68)		29 (17)		137 (82)			100
Nguyen^b^	2018	64				37 (55)	19 (28)	< 130	< 120	70
Rogers^b,d^	2019	99	61 (62)	63 (64)	36 (36)	16 (19)^d^	26 (26)	< 130	< 120	80–90
Sato^c^	2019	69	42 (61)		25 (36)		13 (19)			

All included studies were retrospective cohort studies

^a^n – No. Male = No. Female; n – No. Age ≥ 60 = No. Age < 60; n – No. Osseous flap = No. Soft tissue flap

^b^Authors provided additional data upon contact

^c^Conference proceeding

^d^Hemoglobin data was available for 85 patients

### Mean preoperative Hgb (exposure variable)

**Fig. 2:**
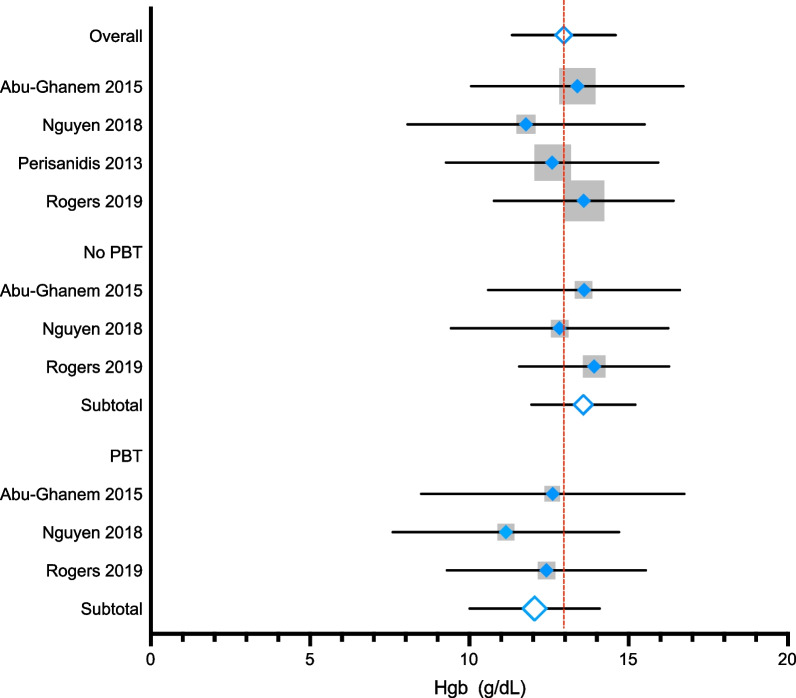
Meta-analysis of preoperative Hgb (g/dL) and subgroup analysis for patients who did not have a perioperative blood transfusion (PBT) and those who did. Hgb, hemoglobin in grams per decaliter; PBT, perioperative blood transfusion

Four studies [32–34, 36] provided mean preoperative Hgb with standard deviations resulting in an overall pooled estimate of 12.96 g/dL (95% CI = 11.33–14.59, I^2^ = 0.00%). When stratified by PBT, the pooled estimate for the no PBT group was 13.58 g/dL (95% CI = 11.95 to 15.21, I^2^ = 0.00%, *p* = 0.875) and was 12.05 g/dL (95% CI = 10.01 to 14.09, I^2^ = 0.00%, *p* = 0.829) for the PBT group (Fig. 2). Two additional studies reported mean preoperative Hgb without standard deviation; an overall weighted mean (weighted by study sample size) including the five studies resulted in similar estimates for the no PBT and PBT groups (13.91 g/dL and 12.37 g/dL, respectively). The mean difference between the PBT and no PBT groups ranged from 0.98 g/dL to 1.76 g/dL.

### Perioperative blood transfusion (outcome variable)

All seven included studies reported the overall proportion of patients exposed to one or more PBTs [11, 21, 32–36]. The overall pooled proportion of patients that received PBT was 41.11% with significant heterogeneity between estimates (95% CI = 17.12–65.11, I^2^ = 98.90%, *p* < 0.01). The proportion of patients that had PBT ranged from 18.84% to 85.21%, representing approximately a fourfold difference between the two extremes. The criteria for PBT varied between studies; three studies reported that PBT was triggered entirely at the discretion of the surgical team whereas the three other studies reported PBT was triggered if preoperative Hgb ranged from 7 to 10 g/dL, while one study did not report the criteria for PBT trigger.

### Sources of heterogeneity

**Fig. 3:**
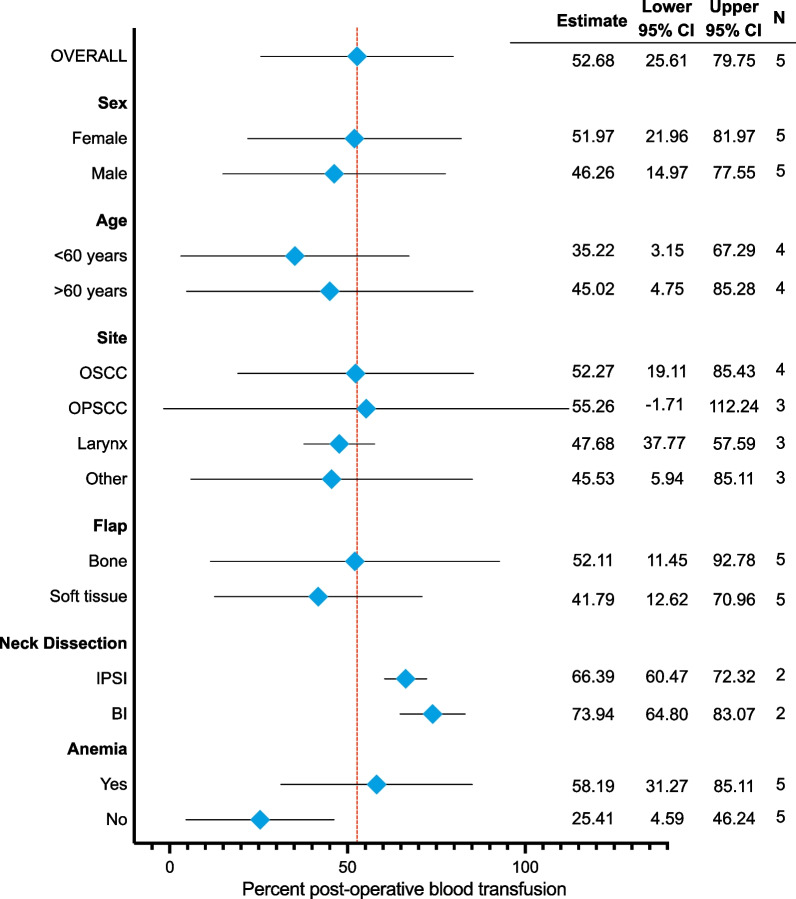
Post-operative blood transfusion by subgroups

Figure 3 shows the sources of heterogeneity explored with regards to the proportion of patients who received PBT.

- *Sex*: The proportion of PBT was reported for each sex (male or female) in five of the included studies [11, 21, 32, 34, 36]. The transfusion rate for males and females was not different (*p* = 0.796).
- *Age*: Four studies [21, 32, 34, 36] reported the proportion of patients above the age of 60 with or without PBT. The proportion of patients over 60 years of age that had a PBT was not different compared to those under 60 years of age (*p* = 0.291).
- *Flap site*: The proportion of patients that had a PBT by tumor site was reported in four studies [11, 21, 32, 34].The tumor sites reported included: oral squamous cell carcinoma (OSCC), oropharyngeal squamous cell carcinoma (OPSCC), the larynx or other sites. The proportion of patients who received a PBT was not different between any site (*p* = 0.983).
- *Flap type*: Five studies [11, 21, 32, 34, 35] reported the proportion of patients receiving PBT with an osseous versus soft tissue flap. The proportion of patients with PBT was not different between the two types of flaps (*p* = 0.856).
- *Neck dissection*: Two studies reported either unilateral or bilateral neck dissection [32, 34]. There was no difference in PBT usage between the two groups (*p* = 0.174).
- *Anemia*: Five studies [21, 32–34, 36] provided the proportion of patients defined as anemic and normal Hgb and their transfusion outcomes (yes or no). There was no difference in PBT among those defined as anemic and among those with normal Hgb (*p* = 0.059). One study was an outlier [32]; when this study was removed from the analysis the transfusion rate was higher among those defined as anemic was (47.62%, 95% CI = 41.19 to 54.06, I^2^ = 0.00%) compared to those with normal Hgb (13.92%, 95% CI = 10.19 to 17.65, I^2^ = 20.69%; *p* < 0.001).

Clinical cancer staging, tumour stage and node stage: We had also pooled to see if there was a difference in PBT exposure between early stage (1 and 2) and late stage (3 and 4) cancer, earlier tumour stages (1 and 2) and advanced tumour stages (3 and 4), as well as the involvement or lack thereof of lymph nodes. In each case, only two studies were available for pooling. The spread of the data within any given subgroup was considered too high to be appropriate for overall pooling estimates.

### Quality and publication bias

Using the Newcastle–Ottawa Scale, four studies [11, 21, 32, 36] received the “good quality” score, which encompassed 3 or 4 stars in the selection, 1 or 2 in comparability and 2 or 3 stars in the outcome domain (Table 2). The other three studies [33–35] received a “poor quality” score, with 0 or 1 stars, 0 stars, or 0 or 1 stars in the selection, comparability, or outcome domains, respectively (Table 2). A funnel plot showed symmetry around the pooled effect size (transfusion rate) depicted with the vertical line but had a large spread of data all outside of the pseudo 95% confidence limits (Additional file 3: Appendix C).

**Table 2:** The Newcastle–Ottawa assessment form for cohort studies for quality appraisal

Study	Representativeness of exposed cohort	Selection of non-exposed cohort	Ascertainment of exposure	Outcome not present at start	Comparability of cohorts	Assessment of outcome	Length of follow-up	Adequacy of follow-up	Quality
Shah	1	1	1	0	1	1	1	1	Good
Perisanidis	1	1	1	0	1	1	1	1	Good
Abu-Ghanem*	1	1	1	0	1	1	1	1	Good
Danan	1	1	1	0	1	1	1	1	Good
Ngyugen	0	0	1	0	0	1	1	1	Poor
Rogers	1	1	1	0	0	1	1	1	Poor
Sato	1	1	1	0	0	0	1	0	Poor

*Ratings based on conference abstract only

## Discussion

This study found significant variation in PBT practices among centers performing HNC surgery with free flap reconstruction. PBT exposure varied by 66% between centers (19% to 85%) and reported transfusion triggers ranged from 7 g/dL to 10 g/dL. The overall pooled PBT rate was 41.11% with significant heterogeneity. We found that patients with low hemoglobin were transfused more than those with normal hemoglobin (47.62% vs 13.92%). Sources for heterogeneity in the proportion of patients receiving PBT between studies were explored and included sex, age over or below 60 years, tumor site, bone or soft tissue flap as well as unilateral or bilateral neck dissection; however, these variables did not explain the heterogeneity between the PBT and no PBT groups. Our review also sought to explore differences in the mean preoperative hemoglobin between those with or without PBT. However, we did not find a difference between the groups.

The findings of this study highlight a two-pronged dilemma related to variance in care delivery. The first dilemma is that it is challenging to identify patients at high risk of PBT. We found that up to 55% of HNC free flap surgery patients present with low preoperative Hgb, however we found substantial variation in the definition of anemia between centers. Notwithstanding guidance by the WHO on clinical values for anemia and low Hgb, it is common that HNC free flap surgery patients undergo HNC surgery with very low preoperative Hgb and without appropriate preoperative interventions. Subsequently, low Hgb results in increased proportion of PBT. We and others have found that, even across various surgical cohorts, low preoperative Hgb increases likelihood of PBT [37–42]. While we did not find a significant difference in mean preoperative Hgb between those who had PBT and those who did not, the highly variable transfusion practices likely contributed to the spread of the data; our weighted mean and mean difference data support this hypothesis. In our center, data show that preoperative Hgb as high as 13 g/dL is associated with a higher risk of PBT (unpublished). Taken together, a new definition for anemia based on patient risk is needed for patients with HNC undergoing major free flap surgery. The purpose of such a definition is to identify, preoperatively, patients at higher risk of PBT and provide treatment prior to surgery.

The second dilemma relates to the appropriateness of PBT triggers. Without a more suitable characterization of anemia, there is a practical challenge in designating appropriate transfusion criteria. We found the rate of PBT varied considerably between studies; with over 80% of patients receiving PBT at some centers [32]. This is related to the varied Hgb thresholds that triggered PBT which was as high as 10 g/dL or 30% hematocrit. Often in free flap surgeries, high transfusion trigger points are arbitrarily established [43]. Recent findings in HNC free flap surgery have shown that a lower PBT trigger (as low as 21% hematocrit or 7 g/dL) can reduce the number of PBT with no significant increase in complications [43, 44]. Transfusion stewardship advocates judicious administration of blood products (i.e. PBT) due to the associated risks to patients and the cost to the healthcare system. While our study was not able to determine the association between mean preoperative Hgb concentration and PBT due to insufficient data, we did find that a high proportion of patients undergoing major HNC free flap surgery receive PBT and that low preoperative Hgb or anemia is likely associated with PBT. Collectively, our findings, taken within the context of the literature, suggest evidence-based and clear transfusion policies are needed. The PBT policies should consider the patient risks associated with PBT, predisposing factors such as preoperative Hgb, and should provide guidelines to manage predisposing factors pre and perioperatively. Both key clinical dilemmas presented have one common element: they can be corrected with action.

Several approaches to managing anemia before surgery have been proposed. Erythropoietin (EPO), preoperative autologous blood donation (PAD) and oral iron are commonly discussed modalities for managing preoperative anemia [16, 23, 33, 45, 46]. However, there are barriers to implementing each of these options. EPO products, while reducing the need of PBT – especially in cancer-related or post radiation/chemotherapy anemia – have raised concerns due to the lack of a clear safety profile [47–49]. PAD, on the other hand, is another way to avoid the immunomodulatory effects of homologous PBT [15, 50]. However, due to the poor cost-effectiveness of this method, as well as the fact that many HNC patients already enter these major surgeries with preoperative anemia, it may be impractical for this cohort [51, 52]. Lastly, oral iron supplementation, is quick, cheap and relatively well-tolerated. On top of patient compliance issues, literature shows, the effectiveness of oral iron can be altered in cancer patients and it may not be an optimal method to prime preoperative hemoglobin levels [53–55], especially when wait times for surgery are less than 6 weeks. Intravenous (IV) iron therapy is a seldom used option that is safe, cost-effective and has been shown to increase blood Hgb concentration where oral iron is ineffective [55, 56]. Blood Hgb can be raised by up to 2 g/dL following IV iron administration in just 3–5 weeks or less [54, 57]. This modest increase in Hgb may be enough to raise Hgb levels above the critical threshold for high-risk preoperative Hgb. Therefore, IV iron is a practical intervention that can be integrated in the time between diagnosis and surgery to increase Hgb and minimize the risk of inappropriate PBT [56, 58]. Effectively, this could reduce both the number of units transfused as well as number of patients receiving PBT. In our review, IV iron was suggested by two studies from the United Kingdom. The National Institute for Health and Care Excellence guidelines in the United Kingdom recommend IV iron for presurgical therapy [34, 59]. This begs the question: are we taking advantage of these technologies to enhance clinical practice among patients undergoing major HNC free flap surgeries?

While this meta-analysis is the first to our knowledge to summarize Hgb and PBT among patients undergoing major HNC free flap surgery, there are a few limitations to consider. We identified a small number of heterogeneous studies, which prevented us from examining all of the predictor variables we would have liked to examine, such as cancer stage, which has been found associated with increased PBT [21, 60, 61]. Similarly, while we aimed to examine the number of units of transfused among those who had a PBT, a potentially important variable, there was insufficient and inconsistent reporting of the number of units transfused to be able to synthesize these data. As is common among systematic reviews, there is a chance that we may have missed unpublished studies in our search strategy; however, we searched grey literature and included conference abstracts to maximize the number of studies included and minimize publication bias (which was not found to be significant).

In conclusion, our systematic review and meta-analysis found that patients with low Hgb are transfused more often than those with normal Hgb undergoing major HNC free flap surgery, but that transfusion practices vary widely. Based on the significant variance in clinical practices around preoperative anemia and PBT found in our study, we advocate for clarity in the definition of anemia and PBT policies. Among patients undergoing major HNC free flap surgeries there is often a 3–6-week window between diagnosis and surgery, which presents an opportunity to initiate interventions to increase Hgb and reduce the need for PBT.

## Supplementary Information

**Additional file 1**. Search strategy.

**Additional file 2**. Data dictionary for the data abstraction form.

**Additional file 3**. Funnel plot to visualize the publication bias.

## Data Availability

The datasets used and analyzed during the current study are available from the corresponding author on reasonable request.
