# Supplementary material for: Preoperative hemoglobin and perioperative blood transfusion in major head and neck surgery: a systematic review and meta-analysis
Source: J Otolaryngol Head Neck Surg. 2023 Jan 24;52:3. doi: 10.1186/s40463-022-00588-4 (PMC9872343; doi:10.1186/s40463-022-00588-4)
Supplement: Supplementary file 1 — Additional file 1. Search strategy. [file 40463_2022_588_MOESM1_ESM.docx]

**Appendix A.** Search Strategy

All searched conducted at 8:13 PM (GMT-6) on February 03, 2020

**Database: MEDLINE (Ovid) <from inception to present>**

**Search strategy:**

1 otolaryngology/ (12844)

2 otorhinolaryngologic surgical procedures/ or laryngectomy/ or laryngoplasty/ or laryngoscopy/ or nasal surgical procedures/ or pharyngectomy/ or pharyngostomy/ or tonsillectomy/ or tracheostomy/ or tracheotomy/ or glossectomy/ or mandibular osteotomy/ or mandibular reconstruction/ or maxillary osteotomy/ (54225)

3 "head and neck neoplasms"/ or otorhinolaryngologic neoplasms/ or ear neoplasms/ or laryngeal neoplasms/ or nose neoplasms/ or pharyngeal neoplasms/ or "squamous cell carcinoma of head and neck"/ or facial neoplasms/ or mouth neoplasms/ or tracheal neoplasms/ or oropharyngeal neoplasms/ or hypopharyngeal neoplasms/ or paranasal sinus neoplasms/ or skull neoplasms/ or orbital neoplasms/ or skull base neoplasms/ (165630)

4 Free Tissue Flaps/ or Neck Dissection/ (11240)

5 jaw neoplasms/ or mandibular neoplasms/ or maxillary neoplasms/ or palatal neoplasms/ or gingival neoplasms/ or salivary gland neoplasms/ or tongue neoplasms/ or maxillary sinus neoplasms/ or parotid neoplasms/ or tonsillar neoplasms/ or sublingual gland neoplasms/ or submandibular gland neoplasms/ (51506)

6 (otolaryng* or otorhinolaryng*).tw,kf. (26678)

7 ((free or myocutaneous or microvascular or microsurgical) adj2 (flap* or graft* or transfer* or reconstruct*)).tw,kf. (26676)

8 ("head and neck" adj3 "squamous cell carcinoma").tw,kf. (13081)

9 (hemi?mandibulectom* or "maxilla?mandibular reconstruct*" or mandibul* or hemi?glossectom* or glossectom* or maxillectom* or laryngectom* or pharyngectom* or pharyngolaryngectom* or palatectom* or hemi?palatectom* or tracheo* or parotidectom* or palatomaxillectom* or hemi?palatomaxillectom* or rhinosurg*).tw,kf. (114436)

10 ((head or neck or "head and neck" or head-and-neck or "head neck" or head-neck or bucc* or scalp* or paranasal or laryn* or pharyn* or hypo?pharyn* or gingiv* or face or mouth or "oral cavity" or jaw or nose or nasal or ear or salivary or tonsil* or mandib* or maxill* or lip* or tongue or "floor of mouth" or "skull base" or skull?base or palat* or craniofacial or intermaxill* or nasopharyn* or intranasal or parotid* or trach* or orbit* or mid?facial or pharyngoesophag* or pharyngolaryng* or gloss* or infra?temporal or aero?digestive or "upper aero?digestive tract" or uadt or maxillofacial or para?pharyn* or submandib* or glotti* or tonsil* or oral or subling* or "salivary duct" or oropharyn*) adj3 (surg* or resect* or dissect* or reconstruct* or osteotom*)).tw,kf. (108757)

11 ((head or neck or "head and neck" or head-and-neck or "head neck" or head-neck or bucc* or scalp* or paranasal or laryn* or pharyn* or hypo?pharyn* or gingiv* or face or mouth or "oral cavity" or jaw or nose or nasal or ear or salivary or tonsil* or mandib* or maxill* or lip* or tongue or "floor of mouth" or "skull base" or skull?base or palat* or craniofacial or intermaxill* or nasopharyn* or intranasal or parotid* or trach* or orbit* or mid?facial or pharyngoesophag* or pharyngolaryng* or gloss* or infra?temporal or aero?digestive or "upper aero?digestive tract" or uadt or maxillofacial or para?pharyn* or submandib* or glotti* or tonsil* or oral or subling* or "salivary duct" or oropharyn*) adj3 (cancer* or tumo?r* or carcinoma* or neoplasm* or metastas* or malignan*)).tw,kf. (179894)

12 1 or 2 or 3 or 4 or 5 or 6 or 7 or 8 or 9 or 10 or 11 (507110)

13 blood transfusion/ or blood transfusion, autologous/ (56334)

14 (blood adj2 transfus*).tw,kf. (57912)

15 ("packed red blood cell*" or "blood product*" or transfusion*).tw,kf. (118292)

16 13 or 14 or 15 (139835)

17 exp anemia/ or hemoglobins/ or hematocrit/ (236039)

18 (an?emi* or h?emoglobin or hematocrit).tw,kf. (297393)

19 17 or 18 (393152)

20 12 and 16 and 19 (317)

**Database: Embase (Ovid) <from inception to present>**

**Search Strategy**

1 otorhinolaryngology/ (22937)

2 ear nose throat surgery/ or parotidectomy/ or throat surgery/ or nose surgery/ or nose reconstruction/ (23225)

3 hypopharynx squamous cell carcinoma/ or larynx squamous cell carcinoma/ or mouth squamous cell carcinoma/ or oropharynx squamous cell carcinoma/ (11081)

4 free tissue graft/ or surgical flaps/ or exp tissue flap/ or exp tissue graft/ (206265)

5 gingiva tumor/ or glossectomy/ or tracheostomy/ or trachea surgery/ or tonsillectomy/ or palatine tonsillectomy/ (39187)

6 oral surgery/ or mandible tumor/ or mandible cancer/ or mandible reconstruction/ or mandible resection/ or mandible osteotomy/ or sagittal split ramal osteotomy/ or maxilla sinus cancer/ or maxilla sinus tumor/ or maxilla sinus carcinoma/ or maxilla tumor/ or maxilla cancer/ or maxilla osteotomy/ or maxilla resection/ (41463)

7 larynx surgery/ or larynx tumor/ or larynx cancer/ or larynx carcinoma/ or larynx granuloma/ or larynx papillomatosis/ or laryngectomy/ (36362)

8 pharynx tumor/ or pharynx cancer/ or pharynx carcinoma.mp. or hypopharynx tumor/ or hypopharynx cancer/ or hypopharynx carcinoma/ or nasopharynx tumor/ or nasopharynx cancer/ or nasopharynx carcinoma/ or oropharynx tumor/ or oropharynx cancer/ or oropharynx carcinoma/ or pharynx polyp/ or pharyngectomy/ (43957)

9 salivary gland tumor/ or salivary gland cancer/ or salivary gland carcinoma/ or parotid gland tumor/ or parotid gland cancer/ or parotid gland carcinoma/ or salivary duct carcinoma/ (18073)

10 "head and neck cancer"/ or eye cancer/ or face cancer/ or "head and neck carcinoma"/ or head cancer/ or jaw cancer/ or lip cancer/ or mouth cancer/ or neck cancer/ or nose cancer/ or orbit cancer/ or paranasal sinus cancer/ or pharynx cancer/ or salivary gland cancer/ or tongue cancer/ or tonsil cancer/ (80379)

11 "head and neck tumor"/ or ear tumor/ or face tumor/ or "head and neck cancer"/ or head tumor/ or jaw tumor/ or lip tumor/ or mouth tumor/ or neck tumor/ or "neoplasms of the eye, lacrimal gland and orbit"/ or nose tumor/ or paranasal sinus tumor/ (90470)

12 "head and neck carcinoma"/ or ameloblastic carcinoma/ or "carcinoma of the eye, lacrimal gland and orbit"/ or "head and neck squamous cell carcinoma"/ or lip carcinoma/ or maxilla sinus carcinoma/ or mouth carcinoma/ or nose carcinoma/ or paranasal sinus carcinoma/ (29914)

13 "head and neck surgery"/ or craniofacial surgery/ or ear surgery/ or neck dissection/ or oral surgery/ (50284)

14 (otolaryng* or otorhinolaryng* or "nasal surgical procedure*" or rhinosurg* or free-flap).tw,kw. (43119)

15 ("head and neck" adj3 "squamous cell carcinoma").tw,kw. (18455)

16 ((free or myocutaneous or microvascular or microsurgical) adj2 (flap* or graft* or transfer* or reconstruct*)).tw,kw. (31026)

17 (hemi?mandibulectom* or "maxilla?mandibular reconstruct*" or mandibul* or hemi?glossectom* or glossectom* or maxillectom* or laryngectom* or pharyngectom* or pharyngolaryngectom* or palatectom* or hemi?palatectom* or tracheo* or parotidectom* or palatomaxillectom* or hemi?palatomaxillectom*).tw,kw. (126521)

18 ((head or neck or "head and neck" or head-and-neck or "head neck" or head-neck or bucc* or scalp* or paranasal or laryn* or pharyn* or hypo?pharyn* or gingiv* or face or mouth or "oral cavity" or jaw or nose or nasal or ear or salivary or tonsil* or mandib* or maxill* or lip or tongue or "floor of mouth" or "skull base" or skull?base or palat* or craniofacial or intermaxill* or nasopharyn* or intranasal or parotid* or trach* or orbit* or mid?facial or pharyngoesophag* or pharyngolaryng* or gloss* or infra?temporal or aero?digestive or "upper aero?digestive tract" or uadt or maxillofacial or para?pharyn* or submandib* or glotti* or tonsil* or oral or subling* or "salivary duct" or oropharyn*) adj3 (surg* or resect* or dissect* or reconstruct* or osteotom*)).tw,kw. (132138)

19 ((head or neck or "head and neck" or head-and-neck or "head neck" or head-neck or bucc* or scalp* or paranasal or laryn* or pharyn* or hypo?pharyn* or gingiv* or face or mouth or "oral cavity" or jaw or nose or nasal or ear or salivary or tonsil* or mandib* or maxill* or lip or tongue or "floor of mouth" or "skull base" or skull?base or palat* or craniofacial or intermaxill* or nasopharyn* or intranasal or parotid* or trach* or orbit* or mid?facial or pharyngoesophag* or pharyngolaryng* or gloss* or infra?temporal or aero?digestive or "upper aero?digestive tract" or uadt or maxillofacial or para?pharyn* or submandib* or glotti* or tonsil* or oral or subling* or "salivary duct" or oropharyn*) adj3 (cancer* or tumo?r* or carcinoma* or neoplasm* or metastas* or malignan*)).tw,kw. (204936)

20 1 or 2 or 3 or 4 or 5 or 6 or 7 or 8 or 9 or 10 or 11 or 12 or 13 or 14 or 15 or 16 or 17 or 18 or 19 (743328)

21 blood transfusion/ or transfusion/ or blood autotransfusion/ (145423)

22 (blood adj2 transfus*).tw,kw. (81610)

23 ("packed red blood cell*" or "blood product*" or transfusion*).tw,kw. (178840)

24 21 or 22 or 23 (228496)

25 exp anemia/ or hematocrit/ (393635)

26 (an?emi* or h?emoglobin or hematocrit).tw,kw. (396703)

27 25 or 26 (587220)

28 20 and 24 and 27 (1692)

**Database: CINAHL (EBSCOhost) <from inception to present>**

**Search strategy:**

(S1 OR S2 OR S3 OR S4 OR S5 OR S6 OR S7 OR S8 PR S9 OR S10) AND (S11 OR S12) AND (S13 OR S14)

TI ( anemi* or anaemi* or hemoglobin or haemoglobin or hematocrit ) OR AB ( anemi* or anaemi* or hemoglobin or haemoglobin or hematocrit )

(MH "Anemia+") OR (MH "Hematocrit")

TI blood n2 transfus* OR AB blood n2 transfus*

(MH "Blood Transfusion") OR (MH "Blood Transfusion, Autologous")

TI ( ((head or neck or "head and neck" or "head-and-neck" or "head neck" or "head-neck" or bucc* or scalp* or paranasal or laryn* or pharyn* or "hypo#pharyn*" or gingiv* or face or mouth or "oral cavity" or jaw or nose or nasal or ear or salivary or tonsil* or mandib* or maxill* or lip or tongue or "floor of mouth" or "skull base" or "skull#base" or palat* or craniofacial or intermaxill* or nasopharyn* or intranasal or parotid* or trach* or orbit* or "mid#facial" or pharyngoesophag* or pharyngol ...

TI ( ((head or neck or "head and neck" or "head-and-neck" or "head neck" or "head-neck" or bucc* or scalp* or paranasal or laryn* or pharyn* or "hypo#pharyn*" or gingiv* or face or mouth or "oral cavity" or jaw or nose or nasal or ear or salivary or tonsil* or mandib* or maxill* or lip or tongue or "floor of mouth" or "skull base" or "skull#base" or palat* or craniofacial or intermaxill* or nasopharyn* or intranasal or parotid* or trach* or orbit* or "mid#facial" or pharyngoesophag* or pharyngol ...

TI ( "hemi#mandibulectom*" or "maxilla#mandibular reconstruct*" or mandibul* or "hemi#glossectom*" or glossectom* or maxillectom* or laryngectom* or pharyngectom* or pharyngolaryngectom* or palatectom* or "hemi#palatectom*" or tracheo* or parotidectom* or palatomaxillectom* or "hemi#palatomaxillectom*" ) OR AB ( "hemi#mandibulectom*" or "maxilla#mandibular reconstruct*" or mandibul* or "hemi#glossectom*" or glossectom* or maxillectom* or laryngectom* or pharyngectom* or pharyngolaryngectom* or p ...

TI ( "squamous cell carcinoma" n3 "head and neck" ) OR AB ( "squamous cell carcinoma" n3 "head and neck" )

TI ( ((free or myocutaneous) n2 (flap* or graft* or transfer*)) ) OR AB ( ((free or myocutaneous) n2 (flap* or graft* or transfer*)) )

(MH "Mandibular Neoplasms") OR (MH "Maxillary Neoplasms") OR (MH "Palatal Neoplasms") OR (MH "Jaw Neoplasms")

(MH "Surgery, Oral") OR (MH "Glossectomy") OR (MH "Gingivectomy") OR (MH "Maxillofacial Prosthesis")

(MH "Surgical Flaps") OR (MH "Perforator Flap")

(MH "Otorhinolaryngologic Neoplasms") OR (MH "Ear Neoplasms") OR (MH "Laryngeal Neoplasms") OR (MH "Nose Neoplasms") OR (MH "Pharyngeal Neoplasms") OR (MH "Squamous Cell Carcinoma of Head and Neck") OR (MH "Head and Neck Neoplasms") OR (MH "Facial Neoplasms") OR (MH "Mouth Neoplasms") OR (MH "Gingival Neoplasms") OR (MH "Salivary Gland Neoplasms") OR (MH "Tongue Neoplasms") OR (MH "Palatal Neoplasms") OR (MH "Lip Neoplasms") OR (MH "Parotid Neoplasms") OR (MH "Hypopharyngeal Neoplasms") OR (MH " ...

(MH "Surgery, Otorhinolaryngologic") OR (MH "Laryngectomy") OR (MH "Neck Dissection") OR (MH "Pharyngectomy") OR (MH "Pharyngostomy") OR (MH "Tonsillectomy") OR (MH "Tracheostomy") OR (MH "Ear Surgery")

**Database: Cochrane Central Register of Controlled Trials [CENTRAL] (Ovid) <from inception to present>**

**Search strategy:**

1 Otolaryngology/ (67)

2 "head and neck neoplasms"/ or carcinoma, squamous cell/ or facial neoplasms/ or eyelid neoplasms/ or mouth neoplasms/ or gingival neoplasms/ or lip neoplasms/ or palatal neoplasms/ or salivary gland neoplasms/ or parotid neoplasms/ or tongue neoplasms/ or otorhinolaryngologic neoplasms/ or ear neoplasms/ or laryngeal neoplasms/ or nose neoplasms/ or paranasal sinus neoplasms/ or maxillary sinus neoplasms/ or pharyngeal neoplasms/ or oropharyngeal neoplasms/ or tonsillar neoplasms/ or tracheal neoplasms/ or jaw neoplasms/ or mandibular neoplasms/ or maxillary neoplasms/ or skull base neoplasms/ (4551)

3 surgical flaps/ or free tissue flaps/ or myocutaneous flap/ or perforator flap/ (1299)

4 oral surgical procedures/ or gingivectomy/ or glossectomy/ or jaw fixation techniques/ or maxillofacial prosthesis implantation/ or oral surgical procedures, preprosthetic/ or orthognathic surgical procedures/ or osteotomy, le fort/ or osteotomy, sagittal split ramus/ (772)

5 orthognathic surgical procedures/ or alveolar bone grafting/ or mandibular osteotomy/ or mandibular reconstruction/ or maxillary osteotomy/ (167)

6 otorhinolaryngologic surgical procedures/ or adenoidectomy/ or laryngectomy/ or nasal surgical procedures/ or neck dissection/ or pharyngectomy/ or pharyngostomy/ or tonsillectomy/ or tracheostomy/ or tracheotomy/ (1873)

7 (otolaryng* or otorhinolaryng* or "nasal surgical procedure*" or rhinosurg* or free-flap).ti,ab,hw. (2116)

8 ("head and neck" adj3 "squamous cell carcinoma").ti,ab,hw. (2036)

9 ((free or myocutaneous or microvascular or microsurgical) adj2 (flap* or graft* or transfer* or reconstruct*)).ti,ab,hw. (856)

10 (hemi?mandibulectom* or "maxilla?mandibular reconstruct*" or mandibul* or hemi?glossectom* or glossectom* or maxillectom* or laryngectom* or pharyngectom* or pharyngolaryngectom* or palatectom* or hemi?palatectom* or tracheo* or parotidectom* or palatomaxillectom* or hemi?palatomaxillectom*).ti,ab,hw. (7975)

11 ((head or neck or "head and neck" or head-and-neck or "head neck" or head-neck or bucc* or scalp* or paranasal or laryn* or pharyn* or hypo?pharyn* or gingiv* or face or mouth or "oral cavity" or jaw or nose or nasal or ear or salivary or tonsil* or mandib* or maxill* or lip or tongue or "floor of mouth" or "skull base" or skull?base or palat* or craniofacial or intermaxill* or nasopharyn* or intranasal or parotid* or trach* or orbit* or mid?facial or pharyngoesophag* or pharyngolaryng* or gloss* or infra?temporal or aero?digestive or "upper aero?digestive tract" or uadt or maxillofacial or para?pharyn* or submandib* or glotti* or tonsil* or oral or subling* or "salivary duct" or oropharyn*) adj3 (surg* or resect* or dissect* or reconstruct* or osteotom*)).ti,ab,hw. (13313)

12 ((head or neck or "head and neck" or head-and-neck or "head neck" or head-neck or bucc* or scalp* or paranasal or laryn* or pharyn* or hypo?pharyn* or gingiv* or face or mouth or "oral cavity" or jaw or nose or nasal or ear or salivary or tonsil* or mandib* or maxill* or lip or tongue or "floor of mouth" or "skull base" or skull?base or palat* or craniofacial or intermaxill* or nasopharyn* or intranasal or parotid* or trach* or orbit* or mid?facial or pharyngoesophag* or pharyngolaryng* or gloss* or infra?temporal or aero?digestive or "upper aero?digestive tract" or uadt or maxillofacial or para?pharyn* or submandib* or glotti* or tonsil* or oral or subling* or "salivary duct" or oropharyn*) adj3 (cancer* or tumo?r* or carcinoma* or neoplasm* or metastas* or malignan*)).ti,ab,hw. (13865)

13 1 or 2 or 3 or 4 or 5 or 6 or 7 or 8 or 9 or 10 or 11 or 12 (36198)

14 blood transfusion/ or blood transfusion, autologous/ (2338)

15 (blood adj2 transfus*).ti,ab,hw. (10630)

16 ("blood product*" or "packed red blood cell*").tw,ab,hw. (2080)

17 14 or 15 or 16 (11562)

18 exp anemia/ or hematocrit/ (6262)

19 (an?emi* or h?emoglobin* or hematocrit).tw,ab,hw. (53972)

20 18 or 19 (54275)

21 13 and 17 and 20 (119)

**Database: Cochrane Database of Systematic Reviews (Ovid) <from inception to present>**

**Search strategy:**

1 (otolaryng* or otorhinolaryng* or "nasal surgical procedure*" or rhinosurg* or free-flap).ti,ab,kw. (2)

2 ("head and neck" adj3 "squamous cell carcinoma").ti,ab,kw. (5)

3 ((free or myocutaneous or microvascular or microsurgical) adj2 (flap* or graft* or transfer* or reconstruct*)).ti,ab,kw. (0)

4 (hemi?mandibulectom* or "maxilla?mandibular reconstruct*" or mandibul* or hemi?glossectom* or glossectom* or maxillectom* or laryngectom* or pharyngectom* or pharyngolaryngectom* or palatectom* or hemi?palatectom* or tracheo* or parotidectom* or palatomaxillectom* or hemi?palatomaxillectom*).ti,ab,kw. (44)

5 ((head or neck or "head and neck" or head-and-neck or "head neck" or head-neck or bucc* or scalp* or paranasal or laryn* or pharyn* or hypo?pharyn* or gingiv* or face or mouth or "oral cavity" or jaw or nose or nasal or ear or salivary or tonsil* or mandib* or maxill* or lip or tongue or "floor of mouth" or "skull base" or skull?base or palat* or craniofacial or intermaxill* or nasopharyn* or intranasal or parotid* or trach* or orbit* or mid?facial or pharyngoesophag* or pharyngolaryng* or gloss* or infra?temporal or aero?digestive or "upper aero?digestive tract" or uadt or maxillofacial or para?pharyn* or submandib* or glotti* or tonsil* or oral or subling* or "salivary duct" or oropharyn*) adj3 (surg* or resect* or dissect* or reconstruct* or osteotom*)).ti,ab,kw. (105)

6 ((head or neck or "head and neck" or head-and-neck or "head neck" or head-neck or bucc* or scalp* or paranasal or laryn* or pharyn* or hypo?pharyn* or gingiv* or face or mouth or "oral cavity" or jaw or nose or nasal or ear or salivary or tonsil* or mandib* or maxill* or lip or tongue or "floor of mouth" or "skull base" or skull?base or palat* or craniofacial or intermaxill* or nasopharyn* or intranasal or parotid* or trach* or orbit* or mid?facial or pharyngoesophag* or pharyngolaryng* or gloss* or infra?temporal or aero?digestive or "upper aero?digestive tract" or uadt or maxillofacial or para?pharyn* or submandib* or glotti* or tonsil* or oral or subling* or "salivary duct" or oropharyn*) adj3 (cancer* or tumo?r* or carcinoma* or neoplasm* or metastas* or malignan*)).ti,ab,kw. (62)

7 1 or 2 or 3 or 4 or 5 or 6 (182)

8 (blood adj2 transfus*).ti,ab,kw. (164)

9 ("blood product*" or "packed red blood cell*").tw,ab,kw. (214)

10 8 or 9 (314)

11 (an?emi* or h?emoglobin* or hematocrit).tw,ab,kw. (1422)

12 7 and 10 and 11 (3)
