# Supplementary material for: Preoperative hemoglobin and perioperative blood transfusion in major head and neck surgery: a systematic review and meta-analysis
Source: J Otolaryngol Head Neck Surg. 2023 Jan 24;52:3. doi: 10.1186/s40463-022-00588-4 (PMC9872343; doi:10.1186/s40463-022-00588-4)
Supplement: Supplementary file 2 — Additional file 2. Data dictionary for the data abstraction form. [file 40463_2022_588_MOESM2_ESM.docx]

**Appendix B**. Standardized Data Extraction Form

Comments on using this data extraction form:

- Maintain consistency in the way you report data and between reviewers
- Indicate that data was not reported, rather than leaving it blank to clarify that you did not forget to extract it
- Data is easily transferable into Excel

**I. Reviewer Information**

| Date form completed (dd/mm/yyyy) | / / |
| --- | --- |
| Reviewer extracting data (note: add additional reviewers if required) | □ Reviewer A  □ Reviewer B |
| Contact details of reviewer extracting data |  |
| Notes |  |

**II. General Information, Methods & Eligibility**

| **Variable** | **Description** | **Possible values** |
| --- | --- | --- |
| ID | Variable assigned to study | Numeric |
| Author | First author of the study | Open text |
| Year | Year of publication | Numeric |
| Title | Title of study | Open text |
| Journal | Pulication journal | Open text |
| Volume | Volume number of the publication | Numeric |
| Issue | Issue number of the publication | Numeric |
| Pages | Page numbers of the publication | Numeric; range |
| Language | Language of the publication | 1 = English 0= Other |
| Type | Type of publication | 2 = Journal article 1 = Conference proceeding  0 = Other |
| Country | Country of investigation | Open text |
| Region | Region of investigation | Open text |
| Institution | Institutional affiliations | Open text |
| Start date | Start date of the study | Numeric |
| End date | End date of the study | Numeric |
| Design | Study design | 6 = RCT  5 = Quasi-random CT  4 = Prospective cohort  3 = Retrospective cohort  2 = Case-control  1 = Case-series (n > 20)  0 = Other |
| COI | Was conflict of interest reported? | 1 = Yes  0 = No |
| Funding | Specify the funding source(s) for this study | Open text |
| Population | Was the population clearly identifiable as head and neck cancer patients receiving free flap reconstruction? (Note: exclusion of esophageal, thyroid and parathyroid carcinomas). | 1 = Yes 0 = No/unclear |
| Exposure | Was the exposure of interest (ie. preoperative hemoglobin or anemia) reported for the population? | 1 = Yes 0 = No/unclear |
|  |  |  |

**III. Primary Exposure (preoperative Hgb or anemia) and Outcome (perioperative blood transfusion) Variables**

| **Variable** | **Description** | **Possible Values** |
| --- | --- | --- |
| Odds Ratio | Odds ratio of receiving PBT given a low preoperative Hgb | Numeric |
| CI lower | Lower bound confidence interval for *or* | Numeric |
| CI upper | Upper bound confidence interval for *or* | Numeric |
| Anemia - male | Hemoglobin (Hgb) concentration [] under which anemia is defined for males (g/dL) | Numeric |
| Anemia - female | [Hgb] under which anemia is defined for females (g/dL) | Numeric |
| Anemia WHO | Is this in line with the World Health Organization’s (WHO) definition of anemia?  (Hgb concentration below 13g/dL in adult males and below 12g/dL in adult, non-pregnant females) | 1 = Yes 0 = No |
|  | Stratified by anemia? | 1 = Yes  0 = No |
|  | Stratification used: | 1 = anemic…  0 = norm… |
| Normal n | Number of patients with normal [Hgb] as defined by the study | Numeric |
| Normal noPBT n | Number of patients with normal [Hgb] as defined by the study in the no PBT group | Numeric |
| Normal PBT n | Number of patients with normal [Hgb] as defined by the study in the PBT group | Numeric |
| Normal p | Proportion of patients with normal [Hgb] as defined by the study | Numeric |
| Normal noPBT p | Number of patients with normal [Hgb] as defined by the study in the no PBT group | Numeric |
| Normal PBT p | Number of patients with normal [Hgb] as defined by the study in the PBT group | Numeric |
| Anemic n | Number of patients with low [Hgb] as defined by the study | Numeric |
| Anemic noPBT n | Number of patients with low [Hgb] as defined by the study in the no PBT group | Numeric |
| Anemic PBT n | Number of patients with low [Hgb] as defined by the study in the PBT group | Numeric |
| Anemic p | Proportion of patients with low [Hgb] as defined by the study | Numeric |
| Anemic noPBT p | Number of patients with low [Hgb] as defined by the study in the no PBT group | Numeric |
| Anemic PBT p | Number of patients with low [Hgb] as defined by the study in the PBT group | Numeric |
| Median hgb | Median preop [Hgb] (g/dL) | Numeric |
| Median hgb IQR 0 | Interquartile range (IQR), quartile 3 (Q3), for *medhgb* (g/dL) | Numeric |
| Median hgb IQR1 | IQR, Q1 for *medhgb* (g/dL) | Numeric |
| Median hgb noPBT | Median preop [Hgb] (g/dL) in the no PBT group | Numeric |
| Median hgb noPBT IQR0 | IQR, Q3 for *medhgbnopbt* (g/dL) | Numeric |
| Median hgb noPBT IQR1 | IQR, Q1 for *medhgbnopbt* (g/dL) | Numeric |
| Median hgb PBT | Median preop [Hgb] (g/dL) in the PBT group | Numeric |
| Median hgb PBT IQR0 | IQR, Q3 for *medhgbpbt* (g/dL) | Numeric |
| Median hgb PBT IQR1 | IQR, Q1 for *medhgbpbt* (g/dL) | Numeric |
| Mean hgb | Mean preop [Hgb] for the whole sample (g/dL) | Numeric |
| hgb SD | SD for *hgb* | Numeric |
| hgb noPBT | Mean preop [Hgb] for the no PBT group (g/dL) | Numeric |
| hgb noPBT SD | SD for *­hgbnopbt* | Numeric |
| hgb PBT | Mean preop [Hgb] for the PBT group (g/dL) | Numeric |
| hgb PBT SD | SD for *­hgbpbt* | Numeric |
| Mean difference | Mean difference for the mean preop [Hgb] in the PBT and no PBT groups (g/dL) | Numeric |
| hgb 1unit | Mean preop [Hgb] for those transfused 1 unit | Numeric |
| hgb 2unit | Mean preop [Hgb] for those transfused 1 unit (g/dL) | Numeric |
| hgb3 unit | Mean preop [Hgb] for those transfused 1 unit (g/dL) | Numeric |
| No PBT | Proportion of patients with no PBT | Numeric |
| PBT | Proportion of patients with PBT | Numeric |
| n PBT | Number of patients with PBT | Numeric |
| 1 PBT p | Proportion of patients transfused 1 unit | Numeric |
| 2 PBT p | Proportion of patients transfused 2 unit | Numeric |
| 3 PBT p | Proportion of patients transfused 3 unit | Numeric |
| 4 PBT p | Proportion of patients transfused 4 unit | Numeric |
| 5 PBT p | Proportion of patients transfused 5 unit | Numeric |
| 1-4 PBT p | Proportion of patients transfused 1-4 units | Numeric |
| PBT median unit | Median units of PBT transfused | Numeric |
| PBT IQRlow | IQR, Q1 for *pbtmed* | Numeric |
| PBT IQRup | IQR, Q3 for *pbtmed* | Numeric |
| PBT trigger | Trigger [Hgb] for initiation of PBT (g/dL) | Numeric |
|  |  |  |
|  |  |  |

**IV. Sample Characteristics & Secondary Exposure Data (ie. for subgroup analysis)**

*Note: Remove or add variables based on the results relevant to the particular study; variables under include those relevant to our review and can be changed*

| **Variable** | **Description** | **Possible Values** |
| --- | --- | --- |
| n | Sample size | Numeric |
| agemean | Mean age of cohort | Numeric |
| agesd | Standard deviation of *agemean* | Numeric |
| agenopbt | Age of patients in the no transfusion (no PBT) group | Numeric |
| agepbt | Age of patients in transfused (PBT) group | Numeric |
|  | Stratify by age? | 1 = Yes  0 = No |
|  | Stratification used: | 1 = over60…  0 = under60… |
| over60n | Number of patients over 60 years of age | Numeric |
| over60nopbtn | Number of patients over 60 years of age in the no PBT group | Numeric |
| over60pbtn | Number of patients over 60 years of age in the PBT group | Numeric |
| over60p | Proportion of patients over 60 years of age | Numeric |
| over60nopbtp | Proportion of patients over 60 years of age in the no PBT group | Numeric |
| over60pbtp | Proportion of patients over 60 years of age in the PBT group | Numeric |
| under60n | Number of patients under 60 years of age | Numeric |
| under60pbtn | Number of patients under 60 years of age in the no PBT group | Numeric |
| under60nopbtn | Number of patients under 60 years of age in the PBT group | Numeric |
| under60p | Proportion of patients under 60 years of age | Numeric |
| under60pbtp | Proportion of patients under 60 years of age in the no PBT group | Numeric |
| under60nopbtp | Proportion of patients under 60 years of age in the PBT group | Numeric |
|  | Stratify by sex? | 1 = Yes  0 = No |
|  | Stratification used: | 1 = male…  0 = female… |
| malen | Number of male patients | Numeric |
| malenopbtn | Number of male patients in the no PBT group | Numeric |
| malepbtn | Number of male patients in the PBT group | Numeric |
| malep | Proportion of male patients | Numeric |
| malenopbtp | Proportion of male patients in the no PBT group | Numeric |
| malepbtp | Proportion of male patients in the PBT group | Numeric |
| femalen | Number of female patients | Numeric |
| femalenopbtn | Number of female patients in the no PBT group | Numeric |
| femalepbtn | Number of female patients in the PBT group | Numeric |
| femalep | Proportion of female patients | Numeric |
| femalenopbtp | Proportion of female patients in the no PBT group | Numeric |
| femalepbtp | Proportion of female patients in the PBT group | Numeric |
|  | Stratify by American Society of Anesthesiologists Class? | 1 = Yes  0 = No |
|  | Stratification used: | 1 = asa34…  0 = asa12… |
| asa12n | Number of patients in American Society of Anesthesiologists (ASA) Class 1 and 2 | Numeric |
| asa12nopbtn | Number of patients in ASA Class 1 and 2 in the no PBT group | Numeric |
| asa12pbtn | Number of patients in ASA Class 1 and 2 in the PBT group | Numeric |
| asa12p | Proportion of patients in ASA Class 1 and 2 | Numeric |
| asa12nopbtp | Proportion of patients in ASA Class 1 and 2 in the no PBT group | Numeric |
| asa12pbtp | Proportion of patients in ASA Class 1 and 2 in the PBT group | Numeric |
| asa34n | Number of patients in ASA Class 3 and 4 | Numeric |
| asa34nopbtn | Number of patients in ASA Class 3 and 4 in the no PBT group | Numeric |
| asa34pbtn | Number of patients in ASA Class 3 and 4 in the PBT group | Numeric |
| asa34p | Proportion of patients in ASA Class 3 and 4 | Numeric |
| asa34nopbtp | Proportion of patients in ASA Class 3 and 4 in the no PBT group | Numeric |
| asa34pbtp | Proportion of patients in ASA Class 3 and 4 in the PBT group | Numeric |
|  | Stratify by squamous cell carcinoma? | 1 = Yes  0 = No |
|  | Stratification used: | 1 = scc…  0 = noscc… |
| sccn | Number of patients with squamous cell carcinoma (SCC) | Numeric |
| sccnopbtn | Number of patients with SCC in the no PBT group | Numeric |
| sccpbtn | Number of patients with SCC in the PBT group | Numeric |
| sccp | Proportion of with SCC | Numeric |
| sccnopbtp | Proportion of patients with SCC in the no PBT group | Numeric |
| sccpbtp | Proportion of patients with SCC in the PBT group | Numeric |
| nosccn | Number of patients with no SCC | Numeric |
| nosccnopbtn | Number of patients with no SCC in the no PBT group | Numeric |
| nosccpbtn | Number of patients with no SCC in the PBT group | Numeric |
| nosccp | Proportion of with no SCC | Numeric |
| nosccnopbtp | Proportion of patients with no SCC in the no PBT group | Numeric |
| nosccpbtp | Proportion of patients with no SCC in the PBT group | Numeric |
|  | Stratify by cancer stage? | 1 = Yes  0 = No |
|  | Stratification used: | 1 = stage34…  0 = stage12… |
| stage12n | Number of patients with cancer stage 1 and 2 (early stage) | Numeric |
| stage12nopbtn | Number of patients in early stage in the no PBT group | Numeric |
| stage12pbtn | Number of patients in early stage in the PBT group | Numeric |
| stage12p | Proportion of patients in early stage | Numeric |
| stage12nopbtp | Proportion of patients in early stage in the no PBT group | Numeric |
| stage12pbtp | Proportion of patients in early stage in the PBT group | Numeric |
| stage34n | Number of patients with cancer stage 3 and 4 (late stage) | Numeric |
| stage34nopbtn | Number of patients in late stage in the no PBT group | Numeric |
| stage34pbtn | Number of patients in late stage in the PBT group | Numeric |
| stage34p | Proportion of patients in late stage | Numeric |
| stage34nopbtp | Proportion of patients in late stage in the no PBT group | Numeric |
| stage34pbtp | Proportion of patients in late stage in the PBT group | Numeric |
|  | Stratify by tumour stage? | 1 = Yes  0 = No |
|  | Stratification used: | 1 = t34… 0 = t12… |
| t12n | Number of patients in tumor stage 1 and 2 (T1/2) | Numeric |
| t12nopbtn | Number of patients T1/2 in the no PBT group | Numeric |
| t12pbtn | Number of patients in T1/2 in the PBT group | Numeric |
| t12p | Proportion of patients in T1/2 | Numeric |
| t12nopbtp | Proportion of patients in T1/2 in the no PBT group | Numeric |
| t12pbtp | Proportion of patients in T1/2 in the PBT group | Numeric |
| t34n | Number of patients in tumor stage 3 and 4 (T3/4) | Numeric |
| t34nopbtn | Number of patients T3/4 in the no PBT group | Numeric |
| t34pbtn | Number of patients in T3/4 in the PBT group | Numeric |
| t34p | Proportion of patients in T3/4 | Numeric |
| t34nopbtp | Proportion of patients in T3/4 in the no PBT group | Numeric |
| t34pbtp | Proportion of patients in T3/4 in the PBT group | Numeric |
|  | Stratify by node stage? | 1 = Yes  0 = No |
|  | Stratification used: | 1 = n1…  0 = n0… |
| n0n | Number of patients with no nodal involvement (N0) | Numeric |
| n0nopbtn | Number of patients in N0 in the no PBT group | Numeric |
| n0pbtn | Number of patients in N0 in the PBT group | Numeric |
| n0p | Proportion of patients in N0 | Numeric |
| n0nopbtp | Proportion of patients in N0 in the no PBT group | Numeric |
| n0pbtp | Proportion of patients in N0 in the PBT group | Numeric |
| n1n | Number of patients with nodal involvement (N1) | Numeric |
| n1nopbtn | Number of patients in N1 in the no PBT group | Numeric |
| n1pbtn | Number of patients in N1 in the PBT group | Numeric |
| n1p | Proportion of patients in N1 | Numeric |
| n1nopbtp | Proportion of patients in N1 in the no PBT group | Numeric |
| n1pbtp | Proportion of patients in N1 in the PBT group | Numeric |
|  | Stratify by tumor site? | 1 = Yes  2 = No |
|  | Stratification used: | 3 = oscc…  2 = opscc…  1 = larynx…  0 = other… |
| osccn | Number of patients with oral squamous cell carcinoma (OSCC) | Numeric |
| osccnopbtn | Number of patients with OSCC in the no PBT group | Numeric |
| osccpbtn | Number of patients with OSCC in the PBT group | Numeric |
| osccp | Proportion of with OSCC | Numeric |
| osccnopbtp | Proportion of patients with OSCC in the no PBT group | Numeric |
| osccpbtp | Proportion of patients with OSCC in the PBT group | Numeric |
| opsccn | Number of patients with oropharyngeal squamous cell carcinoma (OPSCC) | Numeric |
| opsccnopbtn | Number of patients with OPSCC in the no PBT group | Numeric |
| opsccpbtn | Number of patients with OPSCC in the PBT group | Numeric |
| opsccp | Proportion of with OPSCC | Numeric |
| opsccnopbtp | Proportion of patients with OPSCC in the no PBT group | Numeric |
| opsccpbtp | Proportion of patients with OPSCC in the PBT group | Numeric |
| larynxn | Number of patients with laryngeal squamous cell carcinoma (LSCC) | Numeric |
| larynxnopbtn | Number of patients with LSCC in the no PBT group | Numeric |
| larynxpbtn | Number of patients with LSCC in the PBT group | Numeric |
| larynxp | Proportion of with LSCC | Numeric |
| larynxnopbtp | Proportion of patients with LSCC in the no PBT group | Numeric |
| larynxpbtp | Proportion of patients with LSCC in the PBT group | Numeric |
| othersiten | Number of patients with cancer at another site (other) | Numeric |
| othersitenopbtn | Number of patients with other cancer in the no PBT group | Numeric |
| othersitepbtn | Number of patients with other cancer in the PBT group | Numeric |
| othersitep | Proportion of with other cancer | Numeric |
| othersitenopbtp | Proportion of patients with other cancer in the no PBT group | Numeric |
| othersitepbtp | Proportion of patients with other cancer in the PBT group | Numeric |
|  | Stratify by adverse pathology? | 1 = Yes  0 = No |
|  | Stratification used: | 1 = advpath…  0 = noadvpath… |
| advpathn | Number of patients with adverse pathology (path) [defined as presence of 1 or more of: lymphovascular invasion, perineural invasion, extracapsular spread and/or positive margins] | Numeric |
| advpathnopbtn | Number of patients with adverse path in the no PBT group | Numeric |
| advpathpbtn | Number of patients with adverse path in the PBT group | Numeric |
| advpathp | Proportion of with adverse path | Numeric |
| advpathnopbtp | Proportion of patients with adverse path in the no PBT group | Numeric |
| advpathpbtp | Proportion of patients with adverse path in the PBT group | Numeric |
| noadvpathn | Number of patients with no adverse pathology (path) [defined as presence of 1 or more of: lymphovascular invasion, perineural invasion, extracapsular spread and/or positive margins] | Numeric |
| noadvpathnopbtn | Number of patients with no adverse path in the no PBT group | Numeric |
| noadvpathpbtn | Number of patients with no adverse path in the PBT group | Numeric |
| noadvpathp | Proportion of with no adverse path | Numeric |
| noadvpathnopbtp | Proportion of patients with no adverse path in the no PBT group | Numeric |
| noadvpathpbtp | Proportion of patients with no adverse path in the PBT group | Numeric |
|  | Stratify by recurrence? | 1 = Yes  0 = No |
|  | Stratification used: | 1 = recurr…  0 = norecurr… |
| recurrn | Number of patients with cancer recurrence | Numeric |
| recurrnopbtn | Number of patients with recurrence in the no PBT group | Numeric |
| recurrpbtn | Number of patients with recurrence in the PBT group | Numeric |
| recurrp | Proportion of with recurrence | Numeric |
| recurrnopbtp | Proportion of patients with recurrence in the no PBT group | Numeric |
| recurrpbtp | Proportion of patients with recurrence in the PBT group | Numeric |
| norecurrn | Number of patients with no cancer recurrence | Numeric |
| norecurrnopbtn | Number of patients with no recurrence in the no PBT group | Numeric |
| norecurrpbtn | Number of patients with no recurrence in the PBT group | Numeric |
| norecurrp | Proportion of with no recurrence | Numeric |
| norecurrnopbtp | Proportion of patients with no recurrence in the no PBT group | Numeric |
| norecurrpbtp | Proportion of patients with no recurrence in the PBT group | Numeric |
|  | Stratified by operative time? | 1 = Yes  0 = No |
|  | Stratification used: | 1 = optover10…  0 = optunder10… |
| optunder10n | Number of patients with opt <10hrs | Numeric |
| optunder10nopbtn | Number of patients with opt <10hrs in the no PBT group | Numeric |
| optunder10pbtn | Number of patients with opt <10hrs in the PBT group | Numeric |
| optunder10p | Proportion of with opt <10hrs | Numeric |
| optunder10nopbtp | Proportion of patients with opt <10hrs in the no PBT group | Numeric |
| optunder10pbtp | Proportion of patients with opt <10hrs in the PBT group | Numeric |
| optover10n | Number of patients with opt >10hrs | Numeric |
| optover10nopbtn | Number of patients with opt >10hrs in the no PBT group | Numeric |
| optover10pbtn | Number of patients with opt >10hrs in the PBT group | Numeric |
| optover10p | Proportion of patients with opt >10hrs | Numeric |
| optover10nopbtp | Proportion of patients with opt >10hrs in the no PBT group | Numeric |
| optover10pbtp | Proportion of patients with opt >10hrs in the PBT group | Numeric |
|  | Stratified by salvage treatment? | 1 = Yes  0 = No |
|  | Stratification used: | 1 = salvage…  0 = nosalvage… |
| salvagen | Number of patients undergoing salvage surgery | Numeric |
| salvagenopbtn | Number of patients undergoing salvage in the no PBT group | Numeric |
| salvagepbtn | Number of patients undergoing salvage in the PBT group | Numeric |
| salvagep | Proportion of patients undergoing salvage | Numeric |
| salvagenopbtp | Proportion of patients undergoing salvage in the no PBT group | Numeric |
| salvagepbtp | Proportion of patients undergoing salvage in the PBT group | Numeric |
| nosalvagen | Number of patients not undergoing salvage surgery | Numeric |
| nosalvagenopbtn | Number of patients not undergoing salvage in the no PBT group | Numeric |
| nosalvagepbtn | Number of patients not undergoing salvage in the PBT group | Numeric |
| nosalvagep | Proportion of patients not undergoing salvage | Numeric |
| nosalvagenopbtp | Proportion of patients not undergoing salvage in the no PBT group | Numeric |
| nosalvagepbtp | Proportion of patients not undergoing salvage in the PBT group | Numeric |
|  | Stratified by type of flap? | 1 = Yes  0 = No |
|  | Stratification used: | 4 = rff…  3 = fib…  2= alt…  1 = jej…  0 = iliac… |
| rffn | Number of patients receiving the radial forearm flap (RFF) | Numeric |
| rffnopbtn | Number of patients with RFF in the no PBT group | Numeric |
| rffpbtn | Number of patients with RFF in the PBT group | Numeric |
| rffp | Proportion of patients with RFF | Numeric |
| rffnopbtp | Proportion of patients with RFF in the no PBT group | Numeric |
| rffpbtp | Proportion of patients with RFF in the PBT group | Numeric |
| fibn | Number of patients receiving the fibular free flap (FFF) | Numeric |
| fibnopbtn | Number of patients with FFF in the no PBT group | Numeric |
| fibpbtn | Number of patients with FFF in the PBT group | Numeric |
| fibp | Proportion of patients with FFF | Numeric |
| fibnopbtp | Proportion of patients with FFF in the no PBT group | Numeric |
| fibpbtp | Proportion of patients with FFF in the PBT group | Numeric |
| altn | Number of patients receiving the anterolateral thigh (ALT) flap | Numeric |
| altnopbtn | Number of patients with ALT in the no PBT group | Numeric |
| altpbtn | Number of patients with ALT in the PBT group | Numeric |
| altp | Proportion of patients with ALT | Numeric |
| altnopbtp | Proportion of patients with ALT in the no PBT group | Numeric |
| altpbtp | Proportion of patients with ALT in the PBT group | Numeric |
| jejn | Number of patients receiving free jejunal transfer (FJT) | Numeric |
| jejnopbtn | Number of patients with FJT in the no PBT group | Numeric |
| jejpbtn | Number of patients with FJT in the PBT group | Numeric |
| jejp | Proportion of patients with FJT | Numeric |
| jejnopbtp | Proportion of patients with FJT in the no PBT group | Numeric |
| jejpbtp | Proportion of patients with FJT in the PBT group | Numeric |
| iliacn | Number of patients receiving iliac crest flap | Numeric |
| iliacnopbtn | Number of patients with iliac crest flap in the no PBT group | Numeric |
| iliacpbtn | Number of patients with iliac crest flap in the PBT group | Numeric |
| iliacp | Proportion of patients with iliac crest flap | Numeric |
| iliacnopbtp | Proportion of patients with iliac crest flap in the no PBT group | Numeric |
| iliacpbtp | Proportion of patients with iliac crest flap in the PBT group | Numeric |
|  | Stratified by flap composition? | 1 = Yes  0 = No |
|  | Stratification used: | 1 = oflap…  0 = stflap… |
| oflapn | Number of patients receiving an osseous (hard/bony) free flap | Numeric |
| oflapnopbtn | Number of patients with osseous free flap in the no PBT group | Numeric |
| oflappbtn | Number of patients with osseous free flap in the PBT group | Numeric |
| oflapp | Proportion of patients with osseous free flap | Numeric |
| oflapnopbtp | Proportion of patients with osseous free flap in the no PBT group | Numeric |
| oflappbtp | Proportion of patients with osseous free flap in the PBT group | Numeric |
| stflapn | Number of patients receiving a soft tissue [soft] free flap | Numeric |
| stflapnopbtn | Number of patients with soft tissue free flap in the no PBT group | Numeric |
| stflappbtn | Number of patients with soft tissue free flap in the PBT group | Numeric |
| stflapp | Proportion of patients with soft tissue free flap | Numeric |
| stflapnopbtp | Proportion of patients with soft tissue free flap in the no PBT group | Numeric |
| stflappbtp | Proportion of patients with soft tissue free flap in the PBT group | Numeric |
|  | Stratified by neck dissection? | 1 = Yes  0 = No |
|  | Stratification used: | 1 = ndbi…  0 = ndi… |
| ndin | Number of patients with ipsilateral (or one-sided) neck dissection (ND) | Numeric |
| ndinopbtn | Number of patients with ipsilateral ND in the no PBT group | Numeric |
| ndipbtn | Number of patients with ipsilateral ND in the PBT group | Numeric |
| ndip | Proportion of patients with ipsilateral ND | Numeric |
| ndinopbtp | Proportion of patients with ipsilateral ND in the no PBT group | Numeric |
| ndipbtp | Proportion of patients with ipsilateral ND in the PBT group | Numeric |
| ndbn | Number of patients with bilateral (two-sided) neck dissection (ND) | Numeric |
| ndbnopbtn | Number of patients with bilateral ND in the no PBT group | Numeric |
| ndbpbtn | Number of patients with bilateral ND in the PBT group | Numeric |
| ndbp | Proportion of patients with bilateral ND | Numeric |
| ndbnopbtp | Proportion of patients with bilateral ND in the no PBT group | Numeric |
| ndbpbtp | Proportion of patients with bilateral ND in the PBT group | Numeric |
|  | Stratified by flap revision? | 1 = Yes  0 = No |
|  | Stratification used: | 1 = flaprev…  0 = noflaprev… |
| flaprevn | Number of patients with flap revision | Numeric |
| flaprevnopbtn | Number of patients with flap revision in the no PBT group | Numeric |
| flaprevpbtn | Number of patients with flap revision in the PBT group | Numeric |
| flaprevp | Proportion of patients with flap revision | Numeric |
| flaprevnopbtp | Proportion of patients with flap revision in the no PBT group | Numeric |
| flaprevpbtp | Proportion of patients with flap revision in the PBT group | Numeric |
| noflaprevn | Number of patients with no flap revision | Numeric |
| noflaprevnopbtn | Number of patients with no flap revision in the no PBT group | Numeric |
| noflaprevpbtn | Number of patients with flap no revision in the PBT group | Numeric |
| noflaprevp | Proportion of patients with no flap revision | Numeric |
| noflaprevnopbtp | Proportion of patients with no flap revision in the no PBT group | Numeric |
| noflaprevpbtp | Proportion of patients with no flap revision in the PBT group | Numeric |
|  | Stratified by flap revision? | 1 = Yes  0 = No |
|  | Stratification used: | 1 = flapfail…  0 = noflapfail… |
| flapfailn | Number of patients with no flap failure | Numeric |
| flapfailnopbtn | Number of patients with flap failure in the no PBT group | Numeric |
| flapfailpbtn | Number of patients with flap failure in the PBT group | Numeric |
| flapfailp | Proportion of patients with flap failure | Numeric |
| flapfailnopbtp | Proportion of patients with flap failure in the no PBT group | Numeric |
| flapfailpbtp | Proportion of patients with flap failure in the PBT group | Numeric |
| noflapfailn | Number of patients without no flap failure | Numeric |
| noflapfailnopbtn | Number of patients without flap failure in the no PBT group | Numeric |
| noflapfailpbtn | Number of patients without flap failure in the PBT group | Numeric |
| noflapfailp | Proportion of patients without flap failure | Numeric |
| noflapfailnopbtp | Proportion of patients without flap failure in the no PBT group | Numeric |
| noflapfailpbtp | Proportion of patients without flap failure in the PBT group | Numeric |
|  | Stratified by the mortality? | 1 = Yes  0 = No |
|  | Stratification used: | 1 = death…  0 = alive… |
| deathn | Number of deaths | Numeric |
| deathnopbtn | Number of deaths in the no PBT group | Numeric |
| deathpbtn | Number of deaths in the PBT group | Numeric |
| deathp | Proportion of deaths | Numeric |
| deathnopbtp | Proportion of deaths in the no PBT group | Numeric |
| deathpbtp | Proportion of deaths in the PBT group | Numeric |
| aliven | Number alive | Numeric |
| alivenopbtn | Number alive in the no PBT group | Numeric |
| alivepbtn | Number alive in the PBT group | Numeric |
| alivep | Proportion alive | Numeric |
| alivenopbtp | Proportion alive in the no PBT group | Numeric |
| alivepbtp | Proportion alive in the PBT group | Numeric |
|  | Stratified by the Charslon Comorbidity Index? | 1 = Yes  0 = No |
|  | Stratification used: | 1 = charlson0…  0 = charlson1… |
| charlson0n | Number of patients with a Charlson Comorbidity Index (CCI) of 0 | Numeric |
| charlson0nopbtn | Number of patients with CCI of 0 the no PBT group | Numeric |
| charlson0pbtn | Number of patients with CCI of 0 in the PBT group | Numeric |
| charlson0p | Proportion of patients with CCI of 0 | Numeric |
| charlson0nopbtp | Proportion of patients with CCI of 0 in the no PBT group | Numeric |
| charlson0pbtp | Proportion of patients with CCI of 0 in the PBT group | Numeric |
| charlson1n | Number of patients with CCI of >1 | Numeric |
| charlson1nopbtn | Number of patients with CCI of >1 the no PBT group | Numeric |
| charlson1pbtn | Number of patients with CCI of >1 in the PBT group | Numeric |
| charlson1p | Proportion of patients with CCI of >1 | Numeric |
| charlson1nopbtp | Proportion of patients with CCI of >1 in the no PBT group | Numeric |
| charlson1pbtp | Proportion of patients with CCI of >1 in the PBT group | Numeric |
|  | Stratified by the history of smoking? | 1 = Yes  0 = No |
|  | Stratification used: | 1 = smoke1…  0 = smoke0… |
| hxsmoke0n | Number of patients with no history (Hx) of current smoking | Numeric |
| hxsmoke0nopbtn | Number of patients with no Hx current smoking in the no PBT group | Numeric |
| hxsmoke0pbtn | Number of patients with no Hx current smoking in the PBT group | Numeric |
| hxsmoke0p | Proportion of patients with no Hx current smoking | Numeric |
| hxsmoke0nopbtp | Proportion of patients with no Hx current smoking in the no PBT group | Numeric |
| hxsmoke0pbtp | Proportion of patients with no Hx current smoking in the PBT group | Numeric |
| hxsmoke1n | Number of patients with history (Hx) of current smoking | Numeric |
| hxsmoke1nopbtn | Number of patients with Hx current smoking in the no PBT group | Numeric |
| hxsmoke1pbtn | Number of patients with Hx current smoking in the PBT group | Numeric |
| hxsmoke1p | Proportion of patients with Hx current smoking | Numeric |
| hxsmoke1nopbtp | Proportion of patients with Hx current smoking in the no PBT group | Numeric |
| hxsmoke1pbtp | Proportion of patients with Hx current smoking in the PBT group | Numeric |
|  | Stratified by the history of alcohol usage? | 1 = Yes  0 = No |
|  | Stratification used: | 1 = etoh1…  0 = etoh0… |
| hxetoh0n | Number of patients with no history (Hx) of alcohol (EtOH) [defined as former or never drinking] | Numeric |
| hxetoh0nopbtn | Number of patients with no Hx EtOH in the no PBT group | Numeric |
| hxetoh0pbtn | Number of patients with no Hx EtOH in the PBT group | Numeric |
| hxetoh0p | Proportion of patients with no Hx EtOH | Numeric |
| hxetoh0nopbtp | Proportion of patients with no Hx EtOH in the no PBT group | Numeric |
| hxetoh0pbtp | Proportion of patients with no Hx EtOH in the PBT group | Numeric |
| hxetoh1n | Number of patients with history (Hx) of alcohol (EtOH) [defined as current drinker] | Numeric |
| hxetoh1nopbtn | Number of patients with Hx EtOH in the no PBT group | Numeric |
| hxetoh1pbtn | Number of patients with Hx EtOH in the PBT group | Numeric |
| hxetoh1p | Proportion of patients with Hx EtOH | Numeric |
| hxetoh1nopbtp | Proportion of patients with Hx EtOH in the no PBT group | Numeric |
| hxetoh1pbtp | Proportion of patients with Hx EtOH in the PBT group | Numeric |
|  | Stratified by the history of radiation therapy? | 1 = Yes  0 = No |
|  | Stratification used: | 1 = rtx1…  0 = rtx0… |
| rtx0n | Number of patients with no Hx of radiation therapy (RTX) | Numeric |
| rtx0nopbtn | Number of patients with no RTX in the no PBT group | Numeric |
| rtx0pbtn | Number of patients with no RTX in the PBT group | Numeric |
| rtx0p | Proportion of patients with no RTX | Numeric |
| rtx0nopbtp | Proportion of patients with no RTX in the no PBT group | Numeric |
| rtx0pbtp | Proportion of patients with no RTX in the PBT group | Numeric |
| rtx1n | Number of patients with Hx of RTX | Numeric |
| rtx1nopbtn | Number of patients with RTX in the no PBT group | Numeric |
| rtx1pbtn | Number of patients with RTX in the PBT group | Numeric |
| rtx1p | Proportion of patients with RTX | Numeric |
| rtx1nopbtp | Proportion of patients with RTX in the no PBT group | Numeric |
| rtx1pbtp | Proportion of patients with RTX in the PBT group | Numeric |
|  | Stratified by the history of chemotherapy? | 1 = Yes  0 = No |
|  | Stratification used: | 1 = chemo1…  0 = chemo0… |
| chemo0n | Number of patients with no Hx of chemotherapy (CTX) | Numeric |
| chemo0nopbtn | Number of patients with no CTX in the no PBT group | Numeric |
| chemo0pbtn | Number of patients with no CTX in the PBT group | Numeric |
| chemo0p | Proportion of patients with no CTX | Numeric |
| chemo0nopbtp | Proportion of patients with no CTX in the no PBT group | Numeric |
| chemo0pbtp | Proportion of patients with no CTX in the PBT group | Numeric |
| chemo1n | Number of patients with CTX | Numeric |
| chemo1nopbtn | Number of patients with CTX in the no PBT group | Numeric |
| chemo1pbtn | Number of patients with CTX in the PBT group | Numeric |
| chemo1p | Proportion of patients with CTX | Numeric |
| chemo1nopbtp | Proportion of patients with CTX in the no PBT group | Numeric |
| chemo1pbtp | Proportion of patients with CTX in the PBT group | Numeric |
| kaplan0n | Number of patients with a Kaplan-Feinstein comorbidity Index (KFI) of none or mild (0) | Numeric |
|  | Stratified by the Kaplan-Feinstein Index? | 1 = Yes  0 = No |
|  | Stratification used: | 1 = kaplan1…  0 = kaplan0… |
| kaplan0nopbtn | Number of patients with KFI 0 in the no PBT group | Numeric |
| kaplan0pbtn | Number of patients with KFI 0 in the PBT group | Numeric |
| kaplan0p | Proportion of patients with KFI 0 | Numeric |
| kaplan0nopbtp | Proportion of patients with KFI 0 in the no PBT group | Numeric |
| kaplan0pbtp | Proportion of patients with KFI 0 in the PBT group | Numeric |
| kaplan1n | Number of patients with a Kaplan-Feinstein comorbidity Index (KFI) of moderate or severe (1) | Numeric |
| kaplan1nopbtn | Number of patients with KFI 1 in the no PBT group | Numeric |
| kaplan1pbtn | Number of patients with KFI 1 in the PBT group | Numeric |
| kaplan1p | Proportion of patients with KFI 1 | Numeric |
| kaplan1nopbtp | Proportion of patients with KFI 1 in the no PBT group | Numeric |
| kaplan1pbtp | Proportion of patients with KFI 1 in the PBT group | Numeric |
|  | Stratified by the body mass index? | 1 = Yes  0 = No |
|  | Stratification used: | 1 = bmi1…  0 = bmi0… |
| bmi0n | Number of patients with a low body mass index (BMI) (underweight, <18.5 kg/m^2^) | Numeric |
| bmi0nopbtn | Number of patients with low BMI in the no PBT group | Numeric |
| bmi0pbtn | Number of patients with low BMI in the PBT group | Numeric |
| bmi0p | Proportion of patients with low BMI | Numeric |
| bmi0nopbtp | Proportion of patients with low BMI in the no PBT group | Numeric |
| bmi0pbtp | Proportion of patients with low BMI in the PBT group | Numeric |
| bmi1n | Number of patients with a normal or high BMI (>18.5 kg/m^2^) | Numeric |
| bmi1nopbtn | Number of patients with normal or high BMI in the no PBT group | Numeric |
| bmi1pbtn | Number of patients with normal or high BMI in the PBT group | Numeric |
| bmi1p | Proportion of patients with normal or high BMI | Numeric |
| bmi1nopbtp | Proportion of patients with normal or high BMI in the no PBT group | Numeric |
| bmi1pbtp | Proportion of patients with normal or high BMI in the PBT group | Numeric |
|  | Stratified by surgical site infections? | 1 = Yes  0 = No |
|  | Stratification used: | 1 = ssi…  0 = nossi… |
| ssin | Number of patients with surgical site infections (SSI) | Numeric |
| ssinopbtn | Number of patients with SSI in the no PBT group | Numeric |
| ssipbtn | Number of patients with SSI in the PBT group | Numeric |
| ssip | Proportion of patients with SSI | Numeric |
| ssinopbtp | Proportion of patients with SSI in the no PBT group | Numeric |
| ssipbtp | Proportion of patients with SSI in the PBT group | Numeric |
| nossin | Number of patients with no surgical site infections (SSI) | Numeric |
| nossinopbtn | Number of patients with no SSI in the no PBT group | Numeric |
| nossipbtn | Number of patients with no SSI in the PBT group | Numeric |
| nossip | Proportion of patients with no SSI | Numeric |
| nossinopbtp | Proportion of patients with no SSI in the no PBT group | Numeric |
| nossipbtp | Proportion of patients with no SSI in the PBT group | Numeric |
|  | Other data |  |
| opt | Mean operative time (opt) (hrs) for the sample | Numeric |
| optsd | SD for *opt* (hrs) *[add to other variables if reported]* | Numeric |
| optnopbt | Mean opt (hrs) for the no PBT group | Numeric |
| optpbt | Mean opt (hrs) for the PBT group | Numeric |
| eblall | Estimated blood loss (EBL) (mL) for the sample | Numeric |
| eblnopbt | EBL (mL) for the no PBT group | Numeric |
| eblpbt | EBL (mL) for the PBT group | Numeric |
| losall | Mean length of stay (days) in the sample | Numeric |
| losnopbt | Mean length of stay (days) in the no PBT group | Numeric |
| lospbt | Mean length of stay (days) in the PBT group | Numeric |
| alball | Mean preoperative [albumin] for the sample | Numeric |
| albnopbt | Mean preoperative [albumin] for the no PBT group | Numeric |
| albpbt | Mean preoperative [albumin] for the PBT group | Numeric |

**V. Quality Appraisal**

*Note: See the Newcastle-Ottawa Scale (NOS) for cohort studies*

| **Variable** | **Description** | **Possible values** |
| --- | --- | --- |
| represent | Representativeness of exposed cohort | 2 = 2 stars 1 = 1 star 0 = 0 stars |
| selection | Selection of non-exposed cohort | 1 = 1 star 0 = 0 stars |
| exposure | Ascertainment of exposure | 2 = 2 stars 1 = 1 star 0 = 0 stars |
| startoutcome | Demonstration that outcome was not present at start of study | 1 = 1 star 0 = 0 stars |
| compare | Comparability on basis of design or analysis | 2 = 2 stars 1 = 1 star 0 = 0 stars |
| assessoutcome | Assessment of outcome | 2 = 2 stars 1 = 1 star 0 = 0 stars |
| followup1 | Was follow-up long enough for outcomes to occur | 1 = 1 star 0 = 0 stars |
| followup2 | Adequacy of follow up cohorts | 2 = 2 stars 1 = 1 star 0 = 0 stars |
| totalselection | Total points for “selection” (sum of: *represent, selection, exposure, startoutcome*) | Numeric; 0-6 |
| totalcompare | Total points for “comparibility” (sum of: *compare*) | Numeric; 0-2 |
| totalexposure | Total points for “selection” (sum of: *assessoutcome, followup1, followup2*) | Numeric; 0-5 |
| quality | **Final quality score**  **Good quality**: 3 or 4 stars in selection domain AND 1 or 2 stars in comparability domain AND 2  or 3 stars in outcome/exposure domain  **Fair quality**: 2 stars in selection domain AND 1 or 2 stars in comparability domain AND 2 or 3  stars in outcome/exposure domain  **Poor quality**: 0 or 1 star in selection domain OR 0 stars in comparability domain OR 0 or 1 stars  in outcome/exposure domain | Numeric: 2 = good 1 = fair 0 = poor  (can use text) |
