# Supplementary figures and images for: Preoperative hemoglobin and perioperative blood transfusion in major head and neck surgery: a systematic review and meta-analysis
Source: J Otolaryngol Head Neck Surg. 2023 Jan 24;52:3. doi: 10.1186/s40463-022-00588-4 (PMC9872343; doi:10.1186/s40463-022-00588-4)

Funnel plot with pseudo 95% confidence limits

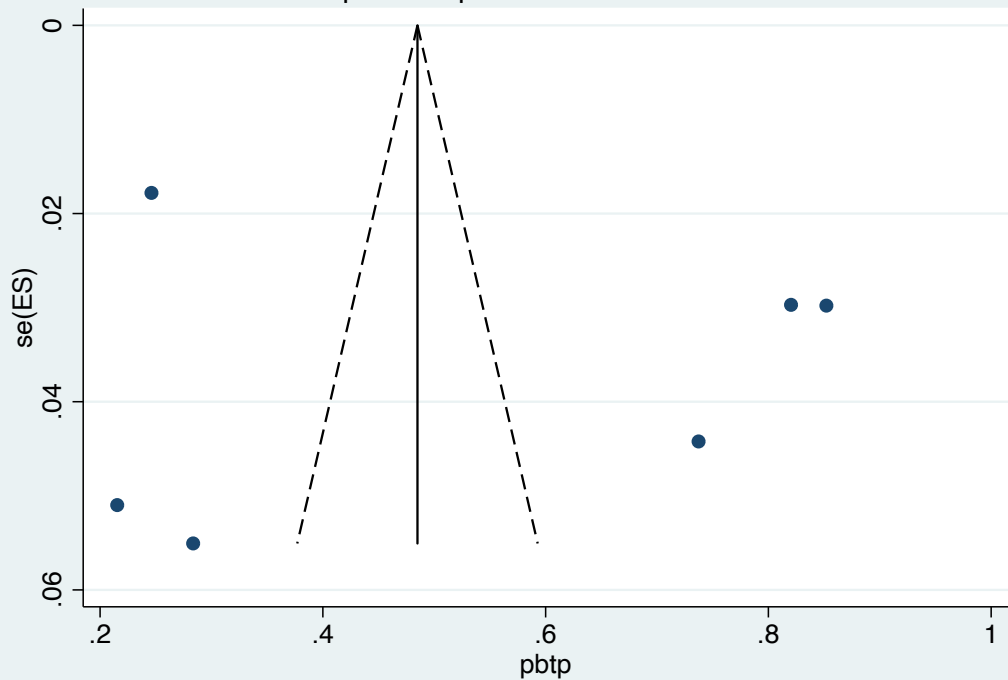

Supplement: Supplementary file 3 — Additional file 3. Funnel plot to visualize the publication bias. [file 40463_2022_588_MOESM3_ESM.pdf]
